# Passive acoustic monitoring can provide insights into occupancy dynamics and impacts of disturbance for at‐risk species

**DOI:** 10.1002/eap.70177

**Published:** 2026-01-15

**Authors:** Jason M. Winiarski, Sheila A. Whitmore, Connor M. Wood, Jonathan P. Eiseman, Erin C. Netoskie, Matthias E. Bieber, H. Anu Kramer, Kevin G. Kelly, Kate McGinn, Craig Thompson, Sarah C. Sawyer, Stefan Kahl, Holger Klinck, M. Zachariah Peery

**Affiliations:** ^1^ Department of Forest and Wildlife Ecology University of Wisconsin–Madison Madison Wisconsin USA; ^2^ K. Lisa Yang Center for Conservation Bioacoustics, Cornell Lab of Ornithology Cornell University Ithaca New York USA; ^3^ Department of Organismal Biology and Ecology Colorado College Colorado Springs Colorado USA; ^4^ USDA Forest Service Pacific Southwest Region Vallejo California USA; ^5^ USDA Forest Service Vallejo California USA; ^6^ Chemnitz University of Technology Chemnitz Germany

**Keywords:** BirdNET, California spotted owl, disturbance, dynamic occupancy model, passive acoustic monitoring, population trends, Sierra Nevada, threatened species

## Abstract

Climate and land‐use change are dramatically altering the frequency, intensity, and extent of ecological disturbances, which threaten the persistence of at‐risk species. To curb the pace and scale of disturbances, balance management and conservation priorities, and alleviate associated population declines, managers require high‐quality information on species' responses to disturbance and their population trends across broad spatial scales that challenge the capacity of traditional, local‐scale monitoring programs. Passive acoustic monitoring is a scalable approach to obtain occurrence data, but the extent to which it can be used to model occupancy dynamics and their environmental drivers remains uncertain. Here, we demonstrate how passive acoustic surveys can be analyzed within a Bayesian dynamic occupancy modeling framework to robustly estimate occupancy dynamics and responses to disturbance in the California spotted owl (*Strix occidentalis occidentalis*), which is threatened by increasingly large, severe “megafires.” From 2021 to 2024, we collected ~2 million hours of audio from autonomous recording units deployed across seven national forests in the Sierra Nevada, California, USA. Spotted owls were less likely to initially occupy and colonize sites that were severely burned, and more likely to go locally extinct following high‐severity fire. Further, we observed declining postfire occupancy trajectories, particularly when sites burned ≥50% high severity. Occupancy trends varied by national forest, but declined by 2% across the entire region. Our findings—which closely align with those from intensive, traditional demographic studies—demonstrate that large‐scale passive acoustic monitoring paired with dynamic occupancy models can effectively detect species' responses to disturbance and estimate population trends, offering valuable insights for management across multiple spatial scales. Finally, we provide specific recommendations to help other passive acoustic monitoring programs successfully detect ecological responses to disturbance and track population changes.

## INTRODUCTION

Biodiversity is declining at unprecedented rates as a consequence of human‐driven climate and land‐use change (Ceballos et al., 2020; Urban, 2015). Increasingly, these changes are altering the frequency, intensity, and extent of biotic and abiotic disturbances (Jones, Abatzoglou, et al., 2022; Weed et al., 2013; Williams et al., 2020), threatening species of concern, ecological communities, and entire ecosystems. For example, milder winters and shifts in growing‐season temperatures and moisture availability have facilitated forest insect and pathogen epidemics globally, with broad consequences for forest composition and wildlife habitat (Seidl et al., 2017; Weed et al., 2013). In addition, synergistic factors such as extreme drought, warming temperatures, and fire suppression have recently fueled exceptionally large and severe “megafires” in a range of biomes worldwide (Bowman et al., 2020), resulting in direct, mass vertebrate mortality (Driscoll et al., 2024; Tomas et al., 2021), persistent population declines (Jones et al., 2021), and extensive ecosystem conversion and habitat loss (Libonati et al., 2020; Steel et al., 2023). Monitoring species' trends and responses to such disturbances across large spatiotemporal scales–and delivery of such information in a timely fashion–is therefore a critical task for guiding biodiversity conservation in an era of rapid environmental change (Lindenmayer, Lavery, & Scheele, 2022; Noon et al., 2012).

The recent development of low‐cost remote sensors such as camera traps and autonomous recording units (ARUs) has transformed our ability to conduct large‐scale ecological monitoring (Gibb et al., 2019; Steenweg et al., 2017). Passive acoustic monitoring (PAM) is an increasingly used tool for biodiversity surveys that allows for noninvasive, high‐resolution data collection via ARU networks spanning regional scales (Darras et al., 2018; Ross et al., 2023; Sethi et al., 2024; Shonfield & Bayne, 2017; Sugai et al., 2019). Innovations in recording hardware have also coincided with a proliferation of state‐of‐the‐art machine‐learning algorithms to automate the detection of sound events and species identification in acoustic data—a process that would be unrealistic otherwise for the terabytes to petabytes of recordings obtained through such networks (Kershenbaum et al., 2025). PAM is thus an increasingly effective and scalable approach for monitoring sound‐producing animals and events at a landscape scale, with a wide range of possible applications including the estimation of wildlife population trends and their drivers (Gibb et al., 2019; Wood et al., 2024).

Detection/non‐detection data generated by ARUs can be analyzed using occupancy models (Balantic & Donovan, 2019; Campos‐Cerqueira & Aide, 2016; Wood et al., 2019), which have undergone extensive development alongside and in response to these emerging conservation technologies (Gilbert et al., 2024; Goldstein et al., 2024). First introduced by MacKenzie et al. (2002), occupancy models estimate species distribution patterns while accounting for imperfect detection with replicated site surveys (MacKenzie et al., 2002; Tyre et al., 2003). While “static” occupancy modeling has been widely applied in PAM studies (e.g., Campos‐Cerqueira & Aide, 2016; Duchac et al., 2021; Hack et al., 2024; Knight et al., 2022; Rugg et al., 2023), such approaches typically rely on data from a single point in time and may fail to capture dynamic processes such as ecological disturbances that shape species occurrence patterns (Miller et al., 2013). The dynamic or multi‐season occupancy model extended the original, single‐season occupancy model to describe changes in occupancy across seasons via colonization and extinction processes (MacKenzie et al., 2003), and can simultaneously accommodate temporal dynamics in occupancy and habitat suitability (Miller et al., 2013). Given their ability to quantify trends in occupancy, identify the mechanisms underlying those changes, and incorporate stochastic disturbance processes, dynamic occupancy models are a powerful tool for PAM and research, with significant potential to improve adaptive management decisions (Balantic & Donovan, 2019) in a rapidly changing world.

Despite these technological and statistical advances, several challenges remain in the implementation of PAM and dynamic occupancy models in a unified framework. First, ARUs are often deployed randomly across a sampling grid and estimate “site” occupancy, which is a more ecologically abstract quantity compared to “territory” occupancy derived under preferential sampling (Weldy et al., 2023; Wood & Peery, 2022). The difference between “site” and “territory” occupancy may increase with home range size because any given ARU becomes more likely to only sample a peripheral part of an individual's home range. Acoustic data collected without prior knowledge of a focal species space use or vocal behavior may pose additional challenges for dynamic occupancy models, where a site “extinction” or “colonization” event might simply represent a change in the area used by an animal or extra‐territorial movement (depending on how a “detection” is defined) rather than the loss or gain in occupancy at an ecologically relevant site (e.g., breeding location; Wood & Peery, 2022). Practically speaking, the observed *site* colonization and extinction rates may be higher than the underlying *territory* colonization and extinction rates. Second, while landscape‐scale monitoring can detect population changes in response to disturbance, accurate estimation of occupancy may not be feasible in the short term to inform adaptive management (e.g., within 5 years of a disturbance event; Wood, 2022; see also Lesmeister et al., 2021). In other cases, landscape‐scale PAM programs may lack the appropriate survey design and statistical power to detect disturbance responses entirely, particularly for rare species or when such programs are established without well‐defined questions and objectives (Lindenmayer & Likens, 2018; Lindenmayer, Lavery, & Scheele, 2022; Lindenmayer, Woinarski, et al., 2022). For example, a large‐scale camera network aimed at monitoring widespread mammals in eastern Australia was unable to capture the effects of severe wildfire on colonization and extinction for rare, disturbance‐sensitive species of conservation concern, which were either not detected or lacked sufficient data to model their responses to the 2019–2020 Black Summer megafires (Lavery et al., 2024). Third, automated species classification algorithms used to analyze large volumes of acoustic data produce false‐positive and false‐negative detections, often requiring the implementation of complex occupancy models that can account for species misclassification (Miller et al., 2011; Rhinehart et al., 2022; Wood et al., 2024; Wright et al., 2020), or semiautomated workflows to reduce manual validation effort and eliminate false‐positives (Campos‐Cerqueira & Aide, 2016; Webber et al., 2022). Consequently, to our knowledge, only a handful of studies exist combining PAM and dynamic occupancy models (e.g., Bielski et al., 2024; Erickson‐Harris et al., 2025; Goodwin et al., 2024; Hofstadter et al., 2022; Law et al., 2024; Rodhouse et al., 2019; Vu et al., 2023; Wood et al., 2020, 2024), and the extent to which this approach can be applied to identify the mechanisms shaping population dynamics remains largely uncertain.

In this study, we demonstrate how audio data from a regional‐scale PAM program can be used in a dynamic occupancy modeling framework to yield robust inferences about ecological responses to disturbance, occupancy dynamics, and population trends in a declining subspecies, the California spotted owl (*Strix occidentalis occidentalis*). The California spotted owl is an iconic old‐forest specialist that has been at the center of a decades‐old debate over forest management in the Sierra Nevada, spurring long‐term demographic monitoring and dozens of studies regarding its ecology, habitat selection, population dynamics, and threats to its viability (Gutiérrez et al., 2017; Verner et al., 1992). In addition to the impacts posed by forest management and an invasive competitor, the barred owl (*S. varia*), recent work—including a comprehensive meta‐analysis—has conclusively shown that spotted owl persistence is threatened by increasingly large, severe wildfires (Jones et al., 2016, 2021; McGinn, Zuckerberg, et al., 2025; McGinn et al., In review; Tempel et al., 2022). In 2021, California spotted owl monitoring transitioned from intensive call‐back and mark‐resight surveys to passive acoustic surveys conducted across the Sierra Nevada bioregion (Kelly et al., 2023); thus, we had the rare opportunity to compare acoustically derived occupancy parameters with those estimated from rigorous long‐term (but spatially limited) studies relying on traditional monitoring methods. Specifically, our objectives were to (1) quantify the ecological drivers of California spotted owl occupancy dynamics: initial occupancy, colonization, and extinction, (2) assess the effects of high‐severity fire on these processes, and (3) estimate trends in annual occupancy at management‐relevant scales (i.e., for the entire Sierra Nevada region, national forests, and wildfire footprints). Our approach—combining passive acoustic surveys, semiautomated species classification, and a dynamic occupancy model—provides an effective way of assessing changes in occupancy and its component processes through space and time, which can guide adaptive management at relevant spatial scales.

## METHODS

### Study area

**FIGURE 1:**
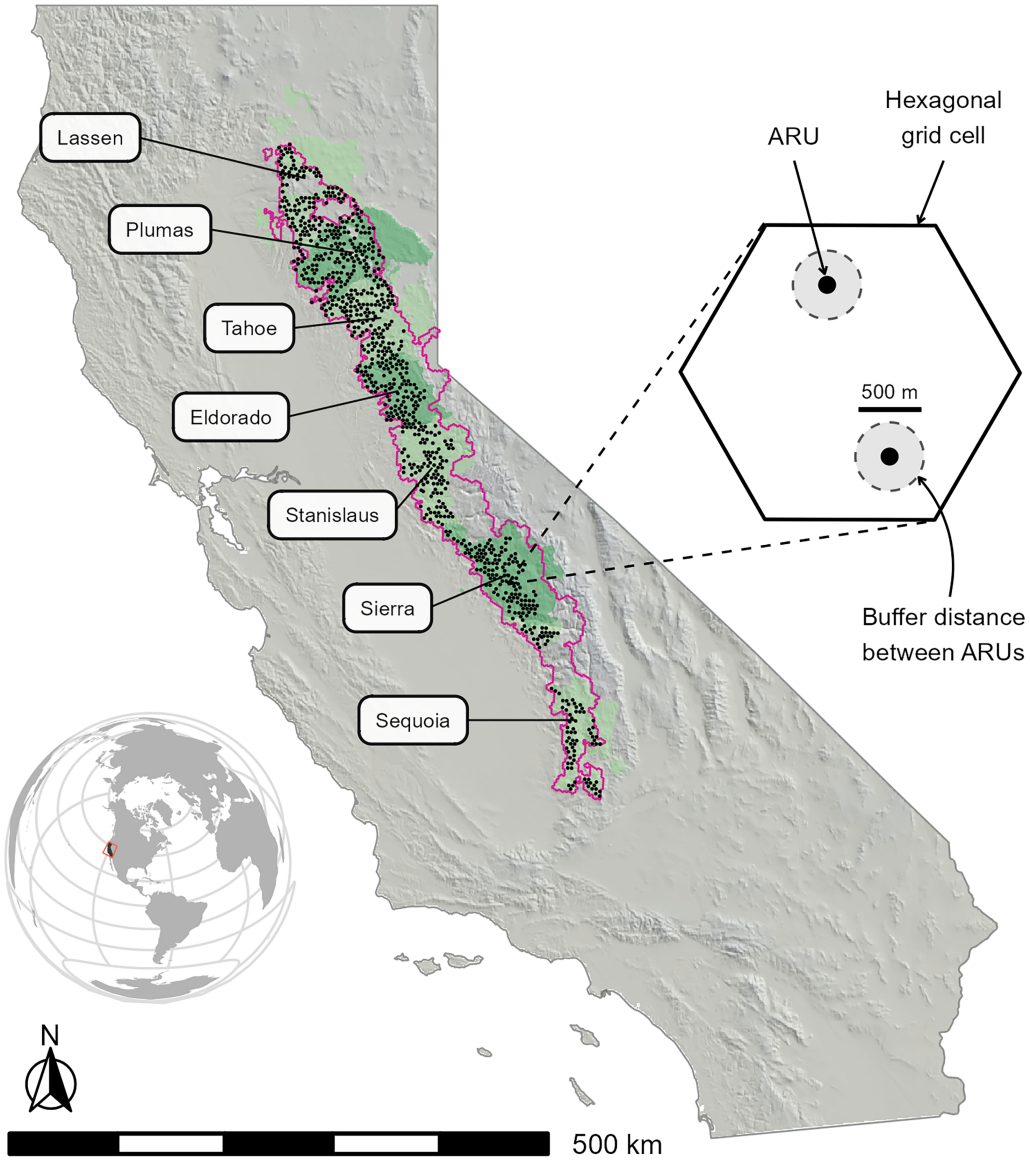
Location of the Sierra Nevada passive acoustic monitoring program in California, USA. The study area (pink border) encompasses suitable spotted owl habitat across seven US national forests (alternating light/dark green polygons) and was divided into a grid of 400‐ha hexagonal cells. Within each surveyed cell, we deployed 1–4 (but generally 2) autonomous recording units (ARUs) ≥ 500 m apart and in acoustically advantageous locations (e.g., ridgetops). Black points represent cells (*n* = 841) surveyed in 2021–2024.

We conducted passive acoustic surveys from 2021 to 2024 as part of an ongoing, long‐term effort to monitor avian biodiversity (>60 species; Brunk, Kramer, et al., 2025; McGinn, Zuckerberg, et al., 2025; Wood et al., 2024) across 3,254,810 ha of foothill and montane forest in eastern California, USA (Figure 1). Our study area encompassed portions of seven national forests, three national parks, and some private lands within the Sierra Nevada and Southern Cascades ecoregions (hereafter, referred to as the Sierra Nevada) and ranged in elevation from 226 to 3985 m. As the sampling design was optimized for monitoring California spotted owl populations, our study area consisted primarily of mixed‐conifer forest on the western slope of the Sierra Nevada (Kelly et al., 2023; Wood et al., 2019), where most spotted owl habitat occurs (Verner et al., 1992). This region is characterized by a Mediterranean climate with warm/dry summers and cool/wet winters, a historic fire regime typified by frequent, low‐ to moderate‐severity fire, and dominant tree species including Douglas‐fir (*Pseudotsuga menziesii*), ponderosa pine (*Pinus ponderosa*), sugar pine (*P. lambertiana*), white fir (*Abies concolor*), and hardwoods such as black oak (*Quercus kelloggii*; North et al., 2016). Forest disturbance events in the Sierra Nevada have recently increased in scale and severity (Ayars et al., 2023; Kramer et al., 2025; Stephens et al., 2022), resulting in extensive habitat degradation and loss. For example, Steel et al. (2023) found a rapid 30% decline in mature forest habitat in the southern Sierra Nevada between 2011 and 2020 due to a combination of high‐severity wildfires, drought, and drought‐associated beetle attacks associated with a warming climate.

### Acoustic sampling

We divided our study area into 8,087,400‐ha hexagonal grid cells, which approximate spotted owl territory size in this region (Tempel, Gutiérrez, et al., 2014). From these, we randomly selected cells for passive acoustic surveys if they (1) did not intersect highways, (2) were ≤50% water, and (3) were accessible from a road. Thus, we excluded cells within particularly steep and wilderness areas, but these accounted for <5% of candidate cells (Kelly et al., 2023). We also avoided surveying adjacent cells to reduce the possibility of double‐counting owl territories (Wood et al., 2019). Because National Park Service lands were not consistently monitored each year, we restricted our analysis to cells within US Forest Service lands.

We typically deployed two ARUs (range = 1–4; Swift or SwiftOne recorder, K. Lisa Yang Center for Conservation Bioacoustics, Cornell Lab of Ornithology, Cornell University, Ithaca, NY, USA) in each selected cell. To maximize sound quality and detection probability, ARUs were deployed at mid‐slope and ridgetop locations with optimal sound propagation, 5–2245 m (mean distance = 34 m) from roads (the vast majority of which were unpaved and rarely traveled) and secured to 15–50 cm (diameter at breast height) trees at a height of 1.5–2 m above the ground. In situ testing and manual review of select audio indicated that spotted owl vocalizations could be detected up to 250 m from an ARU, so whenever possible we deployed ARUs ≥500 m apart and ≥250 m from cell borders (91% of deployments; Figure 1). If a cell overlapped a spotted owl protected activity center (PAC; 121‐ha area of high‐quality habitat around historic spotted owl nest and roost sites), then we attempted to deploy at least one ARU inside the PAC (14% of deployments); otherwise, deployments were made without prior knowledge of owl occupancy. Once selected, we aimed to monitor cells and ARU locations within cells for multiple years, unless access issues such as road closures or safety concerns prevented us from returning to these sites. ARUs were equipped with one omnidirectional microphone with −25 dB sensitivity, ±0.9 V ADC clipping level, and recorded from 18:00 to 09:00 Pacific Daylight Time (PDT) at a sample rate of 32 kHz, 16‐bit resolution, and + 33 dB gain. With these settings, it was possible to collect audio data continuously over a ~5‐week period per ARU deployment. Deployments began in early April, with the latest recordings occurring in early August.

### Spotted owl identification in acoustic data

We subset the raw audio data from 20:00 to 06:00 PDT (i.e., a “survey night”), when spotted owls are most vocally active (Reid et al., 2022). To identify spotted owl vocalizations in the audio files, we used BirdNET (https://github.com/kahst/BirdNET-Analyzer), a deep neural network designed to automate call classification for >6000 species globally, most of them birds (Kahl et al., 2021; Wood & Kahl, 2024). We used a customized version of BirdNET tailored explicitly to our PAM program (i.e., overfit to avian species in our study region and our recording hardware and specifications), and capable of classifying the spotted owl's “four‐note,” “contact,” “crow bark,” “monkey hoot,” and juvenile begging calls. BirdNET analyzes audio data in 3‐s segments, generating a “confidence” score ranging from 0 to 1 for each call type in a segment. While resembling probabilities, confidence scores are a unitless expression of the algorithm's predictive accuracy for a given classification but are generally positively related to each other (Wood & Kahl, 2024). Audio data were processed with BirdNET via the command line and Amazon Cloud Computing.

Following Kelly et al. (2023), we used a confidence score threshold of 0.989 for all spotted owl call types to identify BirdNET predictions as potential detections requiring manual validation. This threshold has been shown to achieve high spotted owl detection probability during the entire ARU sampling period (0.92; Kelly et al., 2023) while ensuring a tractable number of predictions for review. If multiple spotted owl vocalizations with scores ≥0.989 were identified in a 3‐s segment, we kept the vocalization with the highest score. We then manually validated all putative spotted owl detections using Raven Pro sound analysis software (K. Lisa Yang Center for Conservation Bioacoustics, Cornell Lab of Ornithology Center, Ithaca, NY, USA); this was necessary to (1) obtain accurate presence data, which are essential for informing forest management activities that could affect spotted owl habitat quality, and (2) eliminate false‐positive errors, as even moderate incidences of false‐positives can severely bias occupancy and covariate estimates (Berigan et al., 2019; Clare et al., 2021). In our study system, false‐positives can occur when nontarget sounds are misclassified as a spotted owl (e.g., dog barks, cowbells), or when calls broadcast during spotted owl call‐back surveys are identified by BirdNET. Therefore, we also obtained spotted owl survey data from biologists in our study area and discarded any BirdNET predictions that occurred on the same survey night and within 1.5 km of a call‐back survey location (although such incidents were often detected during the manual validation process).

### Data processing and statistical analyses

All data processing and analyses–aside from automated classification with BirdNET and burn severity estimation with Google Earth Engine (Gorelick et al., 2017)–were conducted with R version 4.4.1 (R Core Team, 2024).

#### Environmental variables

We considered four environmental variables that we hypothesized would influence spotted owl occupancy dynamics: latitude, elevation, canopy height, and proportion of a cell burned by high‐severity wildfire (Appendix S1: Table S1). We extracted the northing spatial coordinate (California Albers projection; hereafter referred to as “latitude”) of each cell's centroid. We obtained elevation data at a 10‐m resolution from the 3D Elevation Program Digital Elevation Model (U.S. Geological Survey, 2023) and calculated the mean elevation within each cell. The elevational range of bird species in the Sierra Nevada varies depending on latitudinal position within the mountain range (Brunk et al., 2023; Saracco et al., 2011; Siegel et al., 2011). Therefore, we corrected elevation for geographic location within the Sierra Nevada by using the residuals from a linear regression of elevation on latitude.

We summarized the mean canopy height for each cell from the California Forest Observatory (CFO) state‐wide forest monitoring system (https://forestobservatory.com). CFO uses deep learning algorithms to derive a suite of forest structure metrics at a 10‐m resolution from airborne lidar and satellite imagery (California Forest Observatory, 2023). We used canopy height (CFO model mean absolute error = 1.97 m) from the 2020 CFO dataset, which was predicted from remotely sensed products (i.e., 2019 Sentinel satellite imagery) collected 2 years prior to the initiation of our PAM program.

To quantify high‐severity fire effects, we developed mapped predictions of the composite burn index (CBI; Key & Benson, 2006) within our study area. CBI is a field‐based measurement of fire severity ranging from 0 to 3 (unburned to high severity) and is considered to be a more ecologically meaningful metric of postfire substrate and vegetation change compared to other indices of fire severity (Parks et al., 2019). Parks et al. (2019) developed a random forest model in Google Earth Engine to predict CBI at a 30‐m resolution as a function of several spectral indices derived from Landsat imagery collected 1 year pre‐ and postfire, latitude, and climatic water deficit, which performed well for wildfires in California (𝑅^2^ of observed vs. predicted CBI = 0.73). Following Cova et al. (2023), we applied the Parks et al. CBI model for wildfires ≥4 ha from 1985 to 2023; 1985 is the earliest year possible since Landsat 5 was launched in 1984. First, we obtained wildfire perimeters intersecting our study area from the CAL FIRE Fire and Resource Assessment Program's historical fire perimeters geodatabase and predicted CBI within each fire using the recommended Landsat image window dates for California (1 June–15 September; Parks et al., 2019). Specifically, we used the bias‐corrected version of CBI, which more accurately predicts extreme CBI values (Parks et al., 2019). Next, we discretized bias‐corrected CBI values into categorical fire severity classes, using ≥2.25 as a threshold for defining high‐severity fire (Miller & Thode, 2007). Finally, for each year of acoustic monitoring, we calculated the proportion of each cell burned by high‐severity fire from 1985 to the preceding fire season, which represents a quasi‐permanent loss of habitat (Jones et al., 2016, 2021).

We relied primarily on the *tidyverse*, *sf*, *terra*, and *landscapemetrics* packages (Hesselbarth et al., 2019; Hijmans, 2024; Pebesma, 2018; Wickham et al., 2019) for geospatial data processing.

#### Dynamic occupancy model description

We quantified spotted owl occupancy patterns using a dynamic occupancy model. Dynamic occupancy models estimate the ecological processes of initial occupancy, colonization, and extinction, while accounting for the observation process (MacKenzie et al., 2003). This occupancy modeling framework requires detection/non‐detection data collected at sites using a hierarchical design, in which secondary sampling occasions (i.e., repeat surveys) are nested within primary occasions, such as seasons or years. Occupancy state of a site can change in subsequent primary occasions as a function of colonization and extinction probabilities but is assumed to remain constant within a primary occasion. In our study, we consider each cell as a site (*i*) and primary occasions (*t*) were years, which were divided into 18 week‐long secondary occasions (*j*). We used a stringent definition of occupancy when building detection histories, where true‐positive spotted owl observations across ARU(s) within a cell and secondary occasion were only coded as a 1 (i.e., a detection) when spotted owl observations occurred on ≥2 nights within the corresponding primary occasion (Kelly et al., 2023) to distinguish site occupancy from less ecologically meaningful site use (e.g., extra‐territorial movements) that could violate occupancy model assumptions of site closure and independence (Wood & Peery, 2022). Otherwise, spotted owl observations not meeting our criteria and non‐detections were coded as a 0, and secondary occasions without acoustic monitoring were coded as NAs.

For the observation process, we modeled detection probability (*p*) as a function of survey effort (hours summed across one or more ARUs within a cell), date, and year (categorical effect):

$$\text{logit}(p_{i,t,j})=\alpha_{\left[\mathrm{NF}\right]}+\beta_{1}\times \log{\left(\text{hours}\right)}_{\mathrm{itj}}+\beta_{2}\times \mathrm{dat}e_{\mathrm{itj}}+\beta_{3}\times \mathrm{dat}e_{\mathrm{itj}}^{2}+\beta_{4}\times \mathrm{yea}r_{\mathrm{itj}}.$$

Survey effort was log‐transformed, as we expected detection probability to stabilize once a certain threshold of recording hours per week was reached (Rugg et al., 2023). We included linear and quadratic terms for date to account for a potential peak in calling activity during the breeding season, and a fixed effect of year to accommodate annual variation in detection. We also included a national forest‐level random intercept, α_[NF]_, to account for cell‐level heterogeneity.

Initial occupancy (𝜓_𝑖_) varied spatially as a function of national forest (random intercept), canopy height, elevation, latitude, and the proportion of a cell burned at high‐severity from 1985 to 2020:

$$\text{logit}\left(\psi_{i,2021}\right)=\alpha_{\left[\mathrm{NF}\right]}+\beta_{1}\times \text{canopy}\;\text{heigh}t_{i}+\beta_{2}\times \text{elevatio}n_{i}+\beta_{3}\times \text{elevatio}n_{i}^{2}+\beta_{4}\times \text{latitud}e_{i}+\beta_{5}\times \text{latitud}e_{i}^{2}+\beta_{6}\times \text{proportion}\;\text{high}\;\text{severit}y_{i}.$$

For elevation and latitude, we used linear and quadratic terms as we expected initial occupancy would be most strongly associated with intermediate levels of these variables.

We allowed colonization (𝛾) to vary spatially and temporally:

$$\text{logit}\left(\gamma_{i,t}\right)=\alpha_{\left[\mathrm{NF}\right]}+\beta_{1}\times \text{proportion}\;\text{high}\;\text{severit}y_{\mathrm{it}}+\beta_{2}\times \mathrm{yea}r_{\mathrm{it}},$$

where α_[𝑁𝐹]_ is a random intercept by national forest, 𝛽_1_ is the proportion of a cell burned by high‐severity fire from 1985 to year *t* − 1, and 𝛽_2_ is a fixed, categorical effect of year. We used the same variables in the extinction (𝜖) submodel. Because the most recent CFO forest structure metrics were derived from satellite imagery collected in 2020, canopy height was only used to predict initial occupancy in 2021.

We fit the dynamic occupancy model within a Bayesian framework using the *ubms* package (Kellner et al., 2022) which interfaces with the probabilistic programming language Stan (Carpenter et al., 2017). We used the package's default, weakly informative priors for the model intercept, coefficients, and random effect SD (Kellner et al., 2022), and ran three chains for 1500 iterations each with a warm‐up of 750 iterations. Prior to analysis, all continuous predictors were standardized by centering around the mean and dividing by the SD. Parameter convergence was assessed visually with traceplots, and by evaluating effective sample size (>300) and the $\hat{R}$ statistic for all parameters ($\hat{R}$ ≤ 1.01; Vehtari et al., 2021). We assessed model fit by plotting state and detection submodel residuals (Wright et al., 2019) and from posterior predictive checks of the MacKenzie‐Bailey 𝜒^2^ fit statistic (MacKenzie & Bailey, 2004).

#### Quantifying temporal trends in occupancy

Fitting the dynamic occupancy model in a Bayesian framework enabled us to derive additional metrics of ecological and management interest in a straightforward way. We used the fitted model to estimate spotted owl occupancy probability for the Sierra Nevada region, each national forest, and year by summarizing posterior draws of the projected occupancy trajectory of surveyed grid cells (Kellner et al., 2022; Weir et al., 2009). To examine the effects of recent high‐severity wildfires on spotted owl occupancy, we repeated this procedure, summarizing the projected occupancy of grid cells burned at high severity and reference cells in the same national forest(s) that were not affected by high‐severity fire.

We also quantified trends in occupancy for the Sierra Nevada region and each national forest by calculating the overall mean annual rate of change in occupancy or mean annual growth rate, $\bar{\lambda }$ (Banner et al., 2019; MacKenzie et al., 2017):

$${\bar{\lambda }}_{t}=\frac{\bar{\psi_{t+1}}}{\bar{\psi_{t}}},$$

$$\bar{\lambda }=\frac{1}{T-1}\sum_{t=1}^{T}{\bar{\lambda }}_{t},$$

where ${\bar{\lambda }}_{t}$ is the change in mean occupancy across cells from year 𝑡 to 𝑡 + 1 (calculated per posterior draw), and $\bar{\lambda }$ is the mean of ${\bar{\lambda }}_{t}$ across years (𝑇 − 1). The $\bar{\lambda }$ parameter estimates a linear net change in occupancy, with values less than one indicating a decline in the number of occupied sites (Banner et al., 2019).

## RESULTS

Unless otherwise noted, we present the mean ± SD for all descriptive statistics. To summarize parameter effects and their uncertainty, we provide the mean, 95% credible intervals (CRIs), and the probability of direction (PD). The PD is the proportion of a parameter's posterior distribution that is greater or less than zero and provides an index of effect existence and direction (Makowski et al., 2019). We adapted this metric to evaluate the direction of occupancy trends, where the proportion of $\bar{\lambda }$ posterior samples greater than or less than one represents certainty of a trend being positive or negative, respectively.

### Sampling and detection summary

Our ecosystem‐scale PAM program yielded 1,936,476 h (221 years) of audio recordings from 2021 to 2024 (Table 1). ARU deployments occurred between 7 April and 8 August, and averaged 34 (±7) nights of surveys per ARU. ARUs were deployed between 327 and 2764 m in elevation at 1935 locations, resulting in 841 unique grid cells surveyed across 4 years of monitoring (2.05 ± 0.44 ARUs per cell). Of these cells, 64.3% were monitored in all 3 years, 21.3% in 3 years, 7.4% in 2 years, and 7.0% for a single year (Appendix S1: Figure S1). We manually verified 80,205 BirdNET predictions (i.e., 3‐s audio segments containing putative spotted owl vocalizations at or above the 0.989 confidence score threshold) of which 63,709 were true‐positive spotted owl observations. We saw a marked reduction (mean = 54%) in spotted owl observations in 2023 (*n* = 9730) compared to other years, coinciding with lower survey effort due to a combination of SD card formatting issues (restricted to 86 ARUs in the Stanislaus, Sierra, and Sequoia National Forests) and record snowpack in the Sierra Nevada that hindered our ability to deploy ARUs early in the season and at higher elevations (Table 1).

**TABLE 1:** Summary of passive acoustic monitoring effort and manually confirmed California spotted owl detections, 2021–2024.

National forest	Year	ARUs	Grid cells	Survey hours	Detections
Lassen	2021	235	120	73,157	1172
	2022	230	114	66,329	814
	2023	213	93	71,101	158
	2024	249	119	82,464	485
Plumas	2021	268	147	83,356	1069
	2022	323	144	73,169	1092
	2023	310	131	101,213	1573
	2024	316	139	102,235	2742
Tahoe	2021	235	119	82,255	4121
	2022	197	103	73,433	3970
	2023	170	87	58,750	1608
	2024	181	93	55,180	2196
Eldorado	2021	207	103	71,975	1856
	2022	210	107	72,928	1728
	2023	202	100	71,537	2571
	2024	213	107	69,327	3320
Stanislaus	2021	184	93	62,583	4549
	2022	212	97	72,159	4398
	2023	136	63	43,797	2485
	2024	168	78	61,125	5717
Sierra	2021	222	113	72,965	954
	2022	268	128	94,682	2544
	2023	154	80	39,768	486
	2024	216	106	75,719	2265
Sequoia	2021	125	63	44,532	3566
	2022	176	85	62,992	2989
	2023	139	70	39,534	849
	2024	163	82	58,211	2432
All forests	2021	1476	758	490,823	17,287
	2022	1616	778	515,692	17,535
	2023	1324	624	425,700	9730
	2024	1506	724	504,261	19,157

Abbreviation: ARU, autonomous recording unit.

### Model evaluation

Our model showed adequate convergence with all $\hat{R}$ ≤ 1.01 and effective sample sizes >615 (Appendix S1: Table S2). Plots of submodel residuals and the Bayesian *p*‐value (0.302) from the posterior predictive check also indicated adequate model fit (Appendix S1: Figures S2 and S3).

### Detection process

**FIGURE 2:**
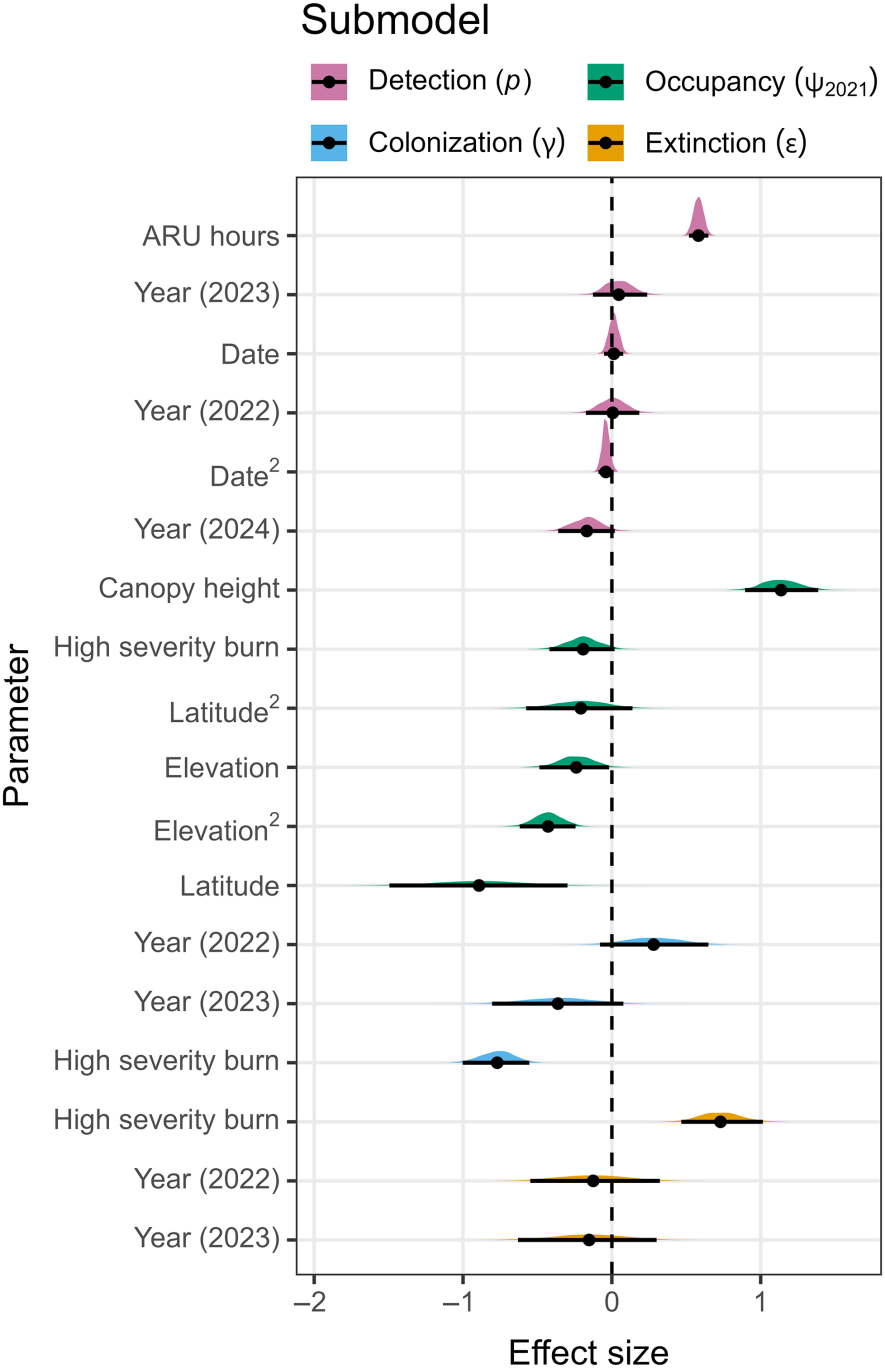
Effect sizes of covariates on detection, initial occupancy, colonization, and extinction. Colored polygons represent full posterior distributions of covariates, while black points and lines represent means and 95% credible intervals. Values greater than zero show a positive effect on the respective observation or ecological process, and mean values lower than zero show a negative effect. Uncertainty in the existence of a positive or negative effect increases with greater overlap of the posterior distribution with zero.

**FIGURE 3:**
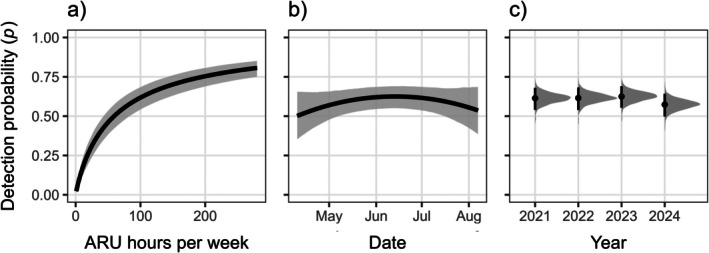
Estimated relationships between detection probability (*p*) and weekly autonomous recording unit (ARU) survey effort (a), date (b), and year (c). Predictions are shown with all other submodel covariates held at their mean value. Black lines represent posterior means and gray shaded areas represent 95% credible intervals (a, b); gray polygons represent full posterior distributions of annual *p* estimates, and black points and lines represent posterior means and 95% credible intervals, respectively (c).

We found a strong, nonlinear relationship (𝛽 = 0.58; 95% CRI = 0.52–0.65; PD = 1.00) between spotted owl detection probability and ARU survey effort, with detection probability sharply increasing then plateauing with additional recording beyond ~100 hours per week (Figure 3a; Appendix S1: Table S2). Date had an uncertain linear effect (𝛽 = 0.01; 95% CRI = −0.05–0.08; PD = 0.65) and a moderate quadratic effect (𝛽 = −0.17; 95% CRI = −0.36–0.02; PD = 0.94), corresponding to a slight peak in detection probability in mid‐June (Figure 3b). Detection probability was lower in 2024 (𝛽 = −0.17; 95% CRI = −0.36–0.02; PD = 0.96) compared to the first 3 years of monitoring (Figures 2 and 3c). Nevertheless, weekly detection probability in 2024 was 0.57 (95% CRI: 0.50–0.64), and the probability of detecting a spotted owl over a typical 5‐week survey period–if present–was high (0.98; 95% CRI: 0.97–0.99).

### Initial occupancy, colonization, and extinction processes

**FIGURE 4:**
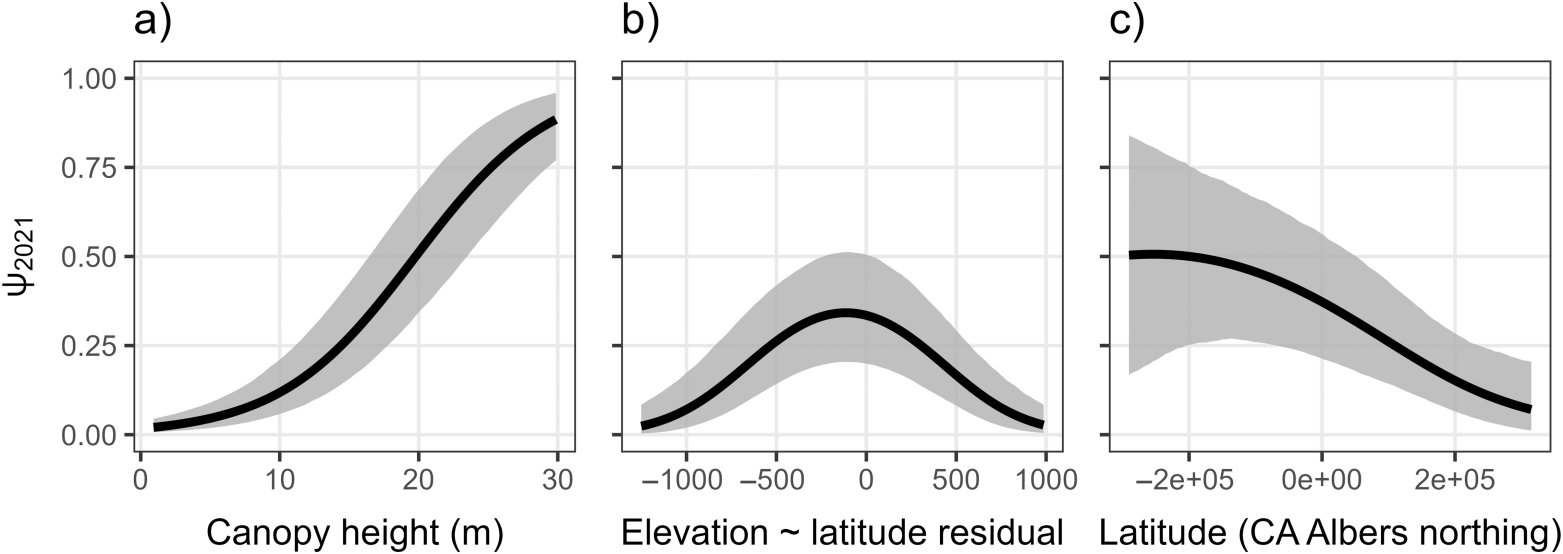
Estimated relationships between canopy height (a), latitude‐corrected elevation (residuals of elevation regressed against latitude; b), latitude (in California Albers projection; c), and initial occupancy probability (𝜓_2021_). Predictions are shown with all other submodel covariates held at their mean value. Black lines represent posterior means and gray shaded areas represent 95% credible intervals.

Mean canopy height was the most important predictor of spotted owl initial occupancy (Figure 2, Appendix S1: Table S2) and had a positive relationship with occupancy probability (𝛽 = 1.14; 95% CRI = 0.90–1.39; PD = 1.00; Figure 4a). There were strong linear (𝛽 = −0.24; 95% CRI = −0.49 to −0.02; PD = 0.98) and quadratic (𝛽 = −0.43; 95% CRI = −0.62 to −0.24; PD = 1.00) effects of elevation after correcting for latitudinal position within the Sierra Nevada, with spotted owls most likely to occur at low‐to‐middle elevations (Figure 4b). Latitude had a strong linear effect (𝛽 = −0.89; 95% CRI = −1.50 to −0.30; PD = 0.99), but a moderate quadratic effect (𝛽 = −0.21; 95% CRI = −0.58 to 0.14; PD = 0.88), suggesting higher occupancy in the southern Sierra Nevada (Figure 4c).

**FIGURE 5:**
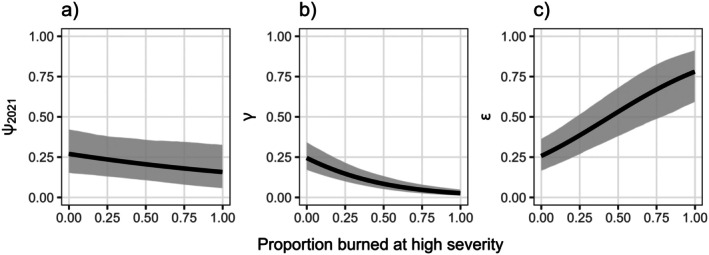
Estimated relationships between the proportion of a cell burned by high‐severity fire and probability of initial occupancy (𝜓_2021_; a), colonization (𝛾; b), and extinction (𝜖; c). Predictions are shown with all other submodel covariates held at their mean value. Black lines represent posterior means and gray shaded areas represent 95% credible intervals.

Spotted owls showed strong, negative relationships with higher proportions of high‐severity fire for initial occupancy (𝛽 = −0.19; 95% CRI = −0.42–0.02; PD = 0.96; Figure 5a) and colonization (𝛽 = −0.77; 95% CRI = −1.00 to −0.56; PD = 1.00; Figure 5b). Conversely, extinction probability increased with a higher proportion of high‐severity fire (𝛽 = 0.73; 95% CRI = 0.47–1.01; PD = 1.00; Figure 5c). There was moderate‐to‐strong support for an effect of year on colonization (𝛽_2022_ = 0.28; 95% CRI = −0.08 to 0.65; PD = 0.93; 𝛽_2023_ = −0.36; 95% CRI = −0.80 to 0.08; PD = 0.95), but the effect was uncertain for the extinction process (𝛽_2022_ = −0.13; 95% CRI = −0.55 to 0.32; PD = 0.71; 𝛽_2023_ = −0.15; 95% CRI = −0.63 to 0.30; PD = 0.73; Figure 2).

### Temporal trends in occupancy

**FIGURE 6:**
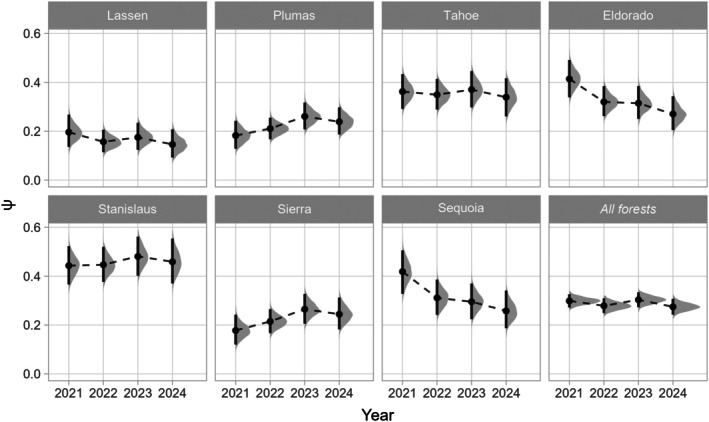
Model‐projected occupancy estimates (𝜓) by national forest and year. Gray polygons represent full posterior distributions of annual 𝜓 estimates, while black points and vertical lines represent means and 95% credible intervals.

**FIGURE 7:**
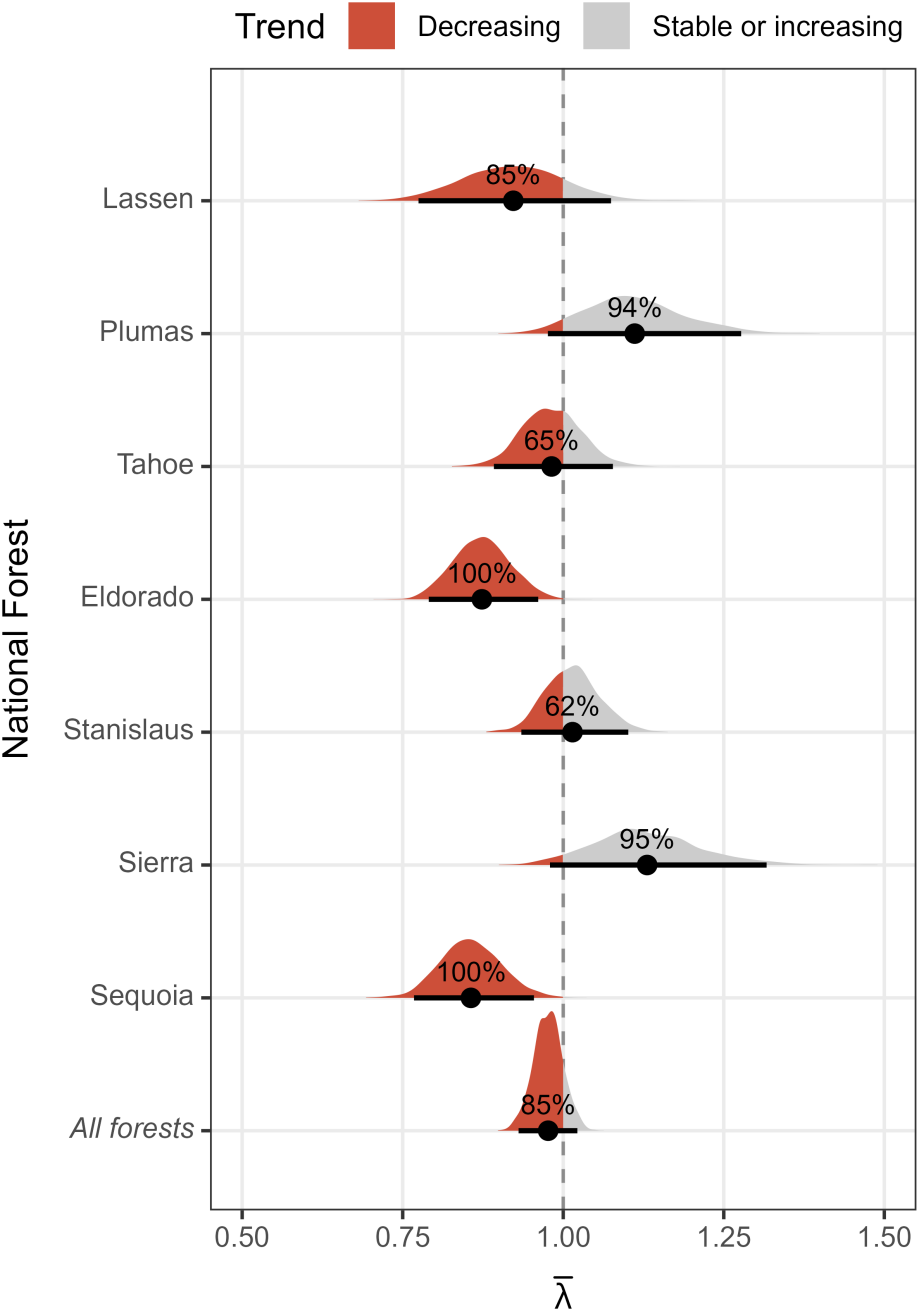
Estimates of net change in occupancy ($\bar{\lambda }$) by national forest from 2021 to 2024. Shaded polygons represent full posterior distributions of $\bar{\lambda }$, while black points and vertical lines represent means and 95% credible intervals. The portion of posterior draws with $\bar{\lambda }$ estimates <1 or ≥ 1 are shown in red and gray, respectively. Labels above mean $\bar{\lambda }$ estimates indicate the percent of posterior draws with a decreasing or stable/increasing trend.

Sierra Nevada‐wide occupancy estimates ranged from 0.30 (95% CRI: 0.27–0.33) in 2023 to 0.27 (95% CRI: 0.24–0.31) in 2024 (Appendix S1: Table S3; Figure 6), and we detected a 2% annual decline in occupancy from 2021 to 2024 ($\bar{\lambda }$: 0.98; 95% CRI: 0.93–1.02; probability of a declining trend: 0.85; Appendix S1: Table S4; Figure 7). Across the entire study period, predicted occupancy was lowest in Lassen National Forest (𝜓_2023_: 0.15; 95% CRI: 0.09–0.21), and highest in Stanislaus National Forest (𝜓_2023_: 0.48; 95% CRI: 0.40–0.56). Trends in occupancy by national forest were variable: Lassen, Eldorado, and Sequoia showed a negative net change in occupancy ($\bar{\lambda }$: 0.86–0.92; probability of declining trend: 0.85–1.00), Plumas and Sierra showed a positive net change in occupancy ($\bar{\lambda }$: 1.11–1.13; probability of a stable or increasing trend: 0.94–0.95), and Tahoe and Stanislaus showed an uncertain net change in occupancy ($\bar{\lambda }$: 0.98–1.01; probability of declining or increasing trend: 0.62–0.65).

**FIGURE 8:**
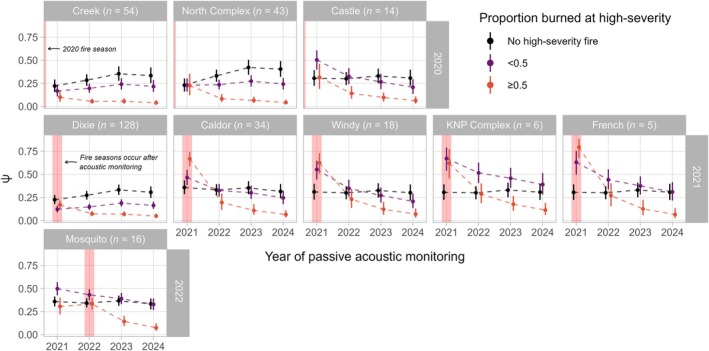
Model‐projected occupancy estimates (𝜓) by wildfire, proportion of cell burned by high‐severity fire, and year of acoustic monitoring. Each panel represents a different wildfire footprint (with number of cells monitored per footprint in parentheses), and panel rows are organized by wildfire ignition year (also indicated by vertical pink shading). Fire categories are unburned at high severity (i.e., reference cells in the same national forest(s) and potentially within a fire perimeter, but not burned at high severity; black), proportion burned less than 0.5 (purple), and proportion burned greater than 0.5 (red). Points represent posterior means and solid lines represent 95% credible interval.

Occupancy of cells burned at high‐severity generally declined in the years following a wildfire (Figure 8). The most significant declines occurred 1 year postfire and were most pronounced when the proportion of a grid cell burned was ≥0.5. Indeed, occupancy estimates of cells more than half burned at high‐severity approached zero by 2024 for every recent wildfire we examined. In contrast, occupancy of reference cells (cells within a wildfire footprint or the surrounding national forest(s) that had not burned at high‐severity from 1985 to 2023) either remained constant or increased slightly in the years following a wildfire.

## DISCUSSION

The emergence of novel disturbance regimes is posing serious challenges for both traditional, local‐scale monitoring approaches and biodiversity conservation. A key question, however, is whether ARU networks and other forms of passive monitoring can provide robust, ecologically meaningful inferences on disturbance effects and population trends to guide adaptive management and conservation (Balantic & Donovan, 2019; Lavery et al., 2024; Wood, 2022; Wood et al., 2019). In this study, we assessed whether detection/non‐detection data from a large‐scale PAM program analyzed within a dynamic occupancy model framework can generate accurate, reliable information on California spotted owl occupancy trends and responses to high‐severity fire at multiple spatial scales within the Sierra Nevada bioregion. We detected the impacts of high‐severity fire on all three processes underlying California spotted owl occupancy dynamics, derived reliable occupancy estimates at multiple spatial scales, and estimated annual trends in occupancy with just 4 years of passive acoustic surveys. Furthermore, by comparing our findings to those of traditional spotted owl demographic studies (detailed below), we provide additional support for the use of PAM paired with dynamic occupancy modeling to reliably inform conservation decision‐making.

Understanding species' responses to disturbance is an increasingly urgent task for PAM programs (Wood, 2022). We found that California spotted owl occupancy dynamics were strongly influenced by high‐severity wildfire: Spotted owls were less likely to initially occupy and colonize grid cells that experienced high‐severity fire, and more likely to go extinct following high‐severity fire (Figure 5). Additionally, the effects of severe wildfire were both immediate (i.e., largely resulting in a sudden drop in occupancy followed by a gradual, continued decline for cells monitored within recent fire perimeters; Figure 8) and long term (i.e., lower initial occupancy of cells burned as early as 1985)—though most high‐severity fire occurred in our study region from approximately 2011 onward (Appendix S1: Figure S4). Wildfires that are atypically large and severe can lead to site extinction via direct mortality and the loss of spotted owl nesting and roosting habitat, which may also prevent recolonization for many decades (Jones, Keyser, et al., 2022; Jones, Abatzoglou, et al., 2022; McGinn, Zuckerberg, et al., 2025). Further, GPS tracking studies have shown that individuals actively avoid foraging within large, severely burned patches (Jones et al., 2020; Kramer et al., 2021; McGinn et al., 2024). We note, however, that cells burned less extensively by high‐severity fire (i.e., proportion of a cell burned <0.5) in a third of the recent fires we examined exhibited stable or slightly increasing postfire occupancy trajectories (Creek, North Complex, and Dixie; Figure 8). GPS‐tagged spotted owls with home ranges overlapping the 2014 King Fire avoided severely burned forest only when >5% of their home range burned at high severity, but continued to occupy home ranges burned up to 40% at high severity in the short term (1–3 years; Jones et al., 2020). Small amounts of high‐severity fire may increase landscape‐scale heterogeneity in vegetation types, which in turn can promote the abundance and consumption of a primary prey species, the dusky‐footed woodrat (*Neotoma fuscipes*; Kuntze et al., 2023). Alternatively, less severely burned areas may provide the only remaining habitat for spotted owls when nearby areas are burned at ≥50% high severity, a threshold associated with spotted owl territory abandonment and direct mortality (Jones et al., 2016, 2021; McGinn et al., In review). Individuals induced to abandon previously occupied territories by fire and/or the loss of a mate may become more mobile and more vocal as they attempt to establish new territories (i.e., in the remaining less severely burned forests), potentially even leading to violations of the site closure assumption that bias occupancy estimates high.

Our findings corroborate previous, multi‐decadal territory occupancy studies demonstrating negative effects of extensive high‐severity fire on California spotted owl occupancy dynamics and the long‐term consequences of this increasing form of disturbance (Jones et al., 2016, 2021; McGinn et al., In review; Tempel et al., 2022). However, by sampling across a large spatial extent (which encompassed cells burned at high severity by 213 fires occurring between 1985 and 2023), we were able to reliably detect responses to severe fire with only 4 years of passive acoustic surveys (Figure 8). Importantly, net negative responses to extensive severe fire likely extend beyond the spotted owl. A recent analysis of passive acoustic surveys from the first year of our study found that the occurrence of 16 bird species was positively associated with spotted owls (Brunk, Kramer, et al., 2025), suggesting that fuels management efforts that support the spotted owl may also benefit these species.

While our results suggest that we accurately estimated the effects of high‐severity fire on colonization and extinction processes, uncertainty remains regarding the ecological realism of these parameters due to the randomized sampling design employed. When randomly deploying ARUs within a survey grid, both colonization and extinction are expected to be larger relative to estimates obtained under preferential sampling (e.g., ARUs deployed at historic breeding locations; Wood & Peery, 2022). Annual colonization probabilities derived from our PAM program (𝛾: 0.12) were slightly lower than those from a recent meta‐analysis of demographic study areas (𝛾: 0.16) by McGinn et al. (In review), but annual extinction probabilities (𝜖: 0.39) were nearly four times higher compared to territory‐based estimates (Appendix S1: Figure S5; 𝜖: 0.10). Higher annual extinction rates in our study may reflect greater survey coverage of occupied sites subsequently affected by high‐severity fire or the inclusion of lower quality habitat compared to demographic surveys. Although the responses to high‐severity fire and occupancy trends that we observed here provide additional support to current ecological understanding, our findings further highlight the nuance required to interpret occupancy parameters derived from passive acoustic surveys (Wood & Peery, 2022). For example, higher extinction rates may be a natural outcome of a site occupancy rather than territory occupancy framework, with some observed site extinctions representing slight movements of territory centers (e.g., in severe fire‐adjacent areas of the Plumas and Sierra National Forests, both of which exhibited increasing occupancy trends)—A process that does not occur with traditional demographic surveys.

Detecting small but ecologically meaningful changes in population size is another major challenge for landscape‐scale PAM programs focused on rare and threatened species (Steenweg et al., 2016; Wood et al., 2019). We detected a 2% annual decline in occupancy ($\bar{\lambda }$: 0.98) across the core range of the California spotted owl in the Sierra Nevada, which is consistent with previously reported estimates of population decline (1%–3%) from three demographic study areas located primarily within national forest land in the Sierra Nevada ($\bar{\lambda }$: 0.97–0.99; Conner et al., 2013; Jones et al., 2018; Tempel, Peery, & Gutiérrez, 2014). While our Sierra Nevada‐wide trend falls within the range of estimates derived from demographic studies, we acknowledge the latter were calculated from long‐term data (~1990–2012) that did not overlap with our study period. Additionally, the probability of a negative trend in occupancy observed in our study was 0.85, but this level of certainty is still fairly high because we expected to detect a ≥2% decline with a 10‐year monitoring program (Wood et al., 2019). Nevertheless, our results suggest that we can rapidly detect ecologically meaningful changes in the Sierra Nevada population of California spotted owls with passive acoustic surveys, and certainty in our trend estimate should increase as additional years of data are collected.

Beyond the Sierra Nevada‐wide occupancy trend estimate, our hierarchical sampling design and Bayesian modeling approach also facilitated the estimation of trends at finer, management‐relevant scales of individual national forests (~320,900–601,700 ha) and wildfire footprints (~10,700–389,400 ha). Forest‐level annual occupancy was estimated with reliable precision ($\bar{\mathrm{CV}}$: 0.12; Figure 6), and we found a high probability of negative or positive trends in occupancy for five national forests (Figure 7). Further, postfire occupancy trajectories derived from our passive acoustic surveys closely reflected those from demographic studies focusing on a single megafire in the central Sierra Nevada (the 2014 King Fire) as well as severe wildfires across the entire bioregion (Jones et al., 2016, 2021; McGinn et al., In review), with sharp drops in occupancy followed by persistent declines for cells that experienced the most high‐severity fire (proportion of a cell burned at high severity ≥0.5), gradual declines for the majority of cells that experienced less high‐severity fire (<0.5 high severity), and generally stable occupancy in cells that never burned at high severity (Figure 8). Our large‐scale monitoring program enables statistical analyses in which information can be shared or pooled across national forests and wildfires via random effects (McElreath, 2020) and increased sample sizes (i.e., by sampling cells within multiple wildfires; Wood, 2022), which may have improved our ability to estimate trends. Simulation‐based power analyses should be conducted to ensure that this and other landscape‐scale PAM programs can robustly detect small but ecologically meaningful changes in occupancy at multiple spatial scales and in response to various drivers (Wood et al., 2019; Wood, 2022).

Our dynamic occupancy modeling approach and inferences regarding population trends rely on several important assumptions that: (1) sites and detections are independent, (2) occupancy status does not change within a primary sampling period (i.e., closure within a season), (3) no false‐positive detections exist, and (4) occupancy is a useful surrogate for population size. To avoid violating occupancy model assumptions, we allowed detection to vary as a function of date, sampled nonadjacent grid cells, limited our passive acoustic surveys to the breeding season, and removed false‐positives through our semiautomated classification pipeline. Previously, by comparing California spotted owl occupancy estimates from our PAM program with spotted owl density estimates from demographic study areas across the Sierra Nevada, we showed that occupancy exhibits a strong, positive relationship with density (Kelly et al., 2023), but this may not be the case in other systems. Although cell occupancy appears to be a reliable indicator of population size, it does not provide information on reproductive status, which may have significant implications for forest management decisions and viability analyses (Appel et al., 2023). Because male and female spotted owl vocalizations are often distinguishable by pitch (Verner et al., 1992), future efforts could leverage our expert‐verified, sex‐specific classifications to estimate pair occupancy using a dynamic multistate occupancy model (MacKenzie et al., 2009). Advances in acoustic individual identification (i.e., distinguishing individuals based on their unique acoustic signature) could also enable the estimation of demographic rates from acoustic recordings in the near future (Knight et al., 2024).

### Limitations, challenges, and future opportunities

We recognize several limitations of our study. First, we relied on somewhat crude (though commonly used) covariates to approximate spotted owl habitat and habitat loss. The CFO canopy height product we used to model initial occupancy was developed from satellite imagery collected 2 years before our first year of monitoring, but fine‐scale annual metrics of forest structure in California are currently lacking. We also assumed that the proportion of a cell burned at high severity resulted in an equivalent loss of habitat, without differentiating between affected habitat types (e.g., nesting/roosting vs. foraging) or considering if the proportion burned included non‐habitat. To refine this metric of habitat change, future work could integrate forest structure metrics to quantify the proportion of high‐severity fire affecting different habitat types. Second, we restricted our analysis to just one form of forest disturbance, despite the prevalence of other disturbance agents in the Sierra Nevada (Steel et al., 2023). In this paper, our goal was to demonstrate the efficacy of large‐scale PAM to detect the effects of disturbance on occupancy dynamics and estimate population trends; however, understanding the effects of additional disturbance types has been a focus of recent research (McGinn, Jones, et al., 2025; Ng et al., 2025). Nevertheless, PAM programs like ours could leverage remote sensing‐based change detection algorithms, such as the Landscape Change Monitoring System (https://apps.fs.usda.gov/lcms-viewer/home.html), to integrate annual forest change from several disturbance types within a dynamic occupancy framework. Third, our reliance on roads for survey access means that any inferences about the spotted owl population in predominantly roadless areas (e.g., designated wilderness areas) represent extrapolation, as opposed to the interpolated inferences made about the accessible areas that were eligible for sampling but remained unsampled based on the initial randomization of our sampling scheme.

The significant investment required to develop a landscape‐scale monitoring program can also create future opportunities in other regions. The successful demonstration of a technique with a high up‐front cost, like large‐scale PAM, can reduce the perceived risk and thus inspire similar efforts in other areas. For example, Gustafson et al. (2025) applied the sampling design of the Sierra Nevada PAM program to the Southern California population segment of the California spotted owl and then refined it based on a combination of initial passive acoustic surveys, regionally specific research on the species movement ecology (McGinn et al., 2024), and Sierra Nevada‐based research on the “vocal home range” of spotted owls (Reid et al., 2022; see also guidance from Rugg et al., 2025 for developing a detection‐based framework that incorporates calling behavior and vocal space use to refine occupancy estimation in PAM). Similarly, multi‐taxa monitoring with an emphasis on spotted owls is being considered in the southwestern United States (Sanderlin et al., 2025). In these and other cases, investments in the field logistics, analytical pipeline, theoretical underpinnings, and natural history for one monitoring program can pay dividends for other efforts.

### Recommendations for PAM programs

Based on our study, which to our knowledge is one of the largest terrestrial acoustic monitoring networks of its kind, we recommend several strategies that may help other PAM programs effectively measure responses to disturbance and track population changes. First, larger sample sizes can increase statistical power to detect population responses to megafires and other severe disturbances (Wood, 2022). By deploying ~1481 ARUs (~724 grid cells) per year, we were more likely to sample within multiple fire footprints and capture a gradient of fire severity from unburned to completely burned at high severity (and with pre‐ and postfire data). Second, deploying ARUs for longer durations (along with large sample sizes) can maximize detection probability, which increases the precision of occupancy estimates and the power to detect trends (Steenweg et al., 2016; Wood et al., 2019). In our study, detection probabilities were high (>0.57), particularly over the course of a typical 5‐week ARU deployment (>0.98). Third, we emphasize conducting passive acoustic surveys across multiple years and keeping ARUs at the same locations from year to year, which can further increase power to detect occupancy trends (Steenweg et al., 2016; Wood et al., 2019). Our program aims to monitor avian biodiversity in the Sierra Nevada over the long term, and we deploy ARUs at permanently established sites unless megafires, record snowpack, and erosion cause road closures that impede access. Although these aspects of our PAM program likely contributed to the successes demonstrated here, we recognize that other factors may have been important, including the pronounced responses of California spotted owls to high‐severity fire, and the ability of our classifier to detect and correctly identify spotted owl vocalizations. Finally, while our bioacoustic data processing pipeline for the spotted owl took several years to refine, it has proven to be transferable to a large suite of species (Brunk, Kramer, et al., 2025; Brunk, Goldberg, et al., 2025; McGinn, Zuckerberg, et al., 2025). For smaller bodied species, the hierarchical sampling that was designed for spotted owls (i.e., sampling grid cells with pairs of ARUs) can be adapted via minor statistical adjustments (e.g., treating each ARU as a sample and including cell‐level random effects to account for potential nonindependence; Brunk et al., 2023). Further, the issues surrounding site versus territory occupancy rates (Wood & Peery, 2022) may decrease due to the greater ratio of an ARU's listening radius to an individual's home range. Ultimately, advances in automated detection of vast audio datasets now allow substantial investments focused on monitoring prominent, disturbance‐sensitive species to be readily expanded into broader biodiversity monitoring programs.

To assist other researchers considering similar large‐scale PAM applications, we provide further details on our program's personnel, data storage, and analysis requirements in Appendix S1: Table S5.

## CONCLUSIONS

Ecosystems are undergoing dramatic changes as a consequence of increasingly severe and frequent ecological disturbances. As biodiversity continues to decline in the face of these global changes, managers and decision‐makers urgently need high‐quality information on species' responses to disturbance and their population trends to curb the pace and scale of associated population declines and losses. Our results and qualitative comparison of estimates derived from our PAM program and traditional demographic studies demonstrate that landscape‐scale PAM can accurately identify the effects of disturbance and provide reliable estimates of occupancy dynamics and trends at multiple, management‐relevant spatial scales—all within a short (~5 year) timeframe. Recent work also suggests that California spotted owl responses to disturbance from fuels management activities, which are critical for reducing fire severity and promoting the long‐term resilience of western US dry forests (Stephens et al., 2020), can also be detected with this monitoring program despite their smaller footprint and more subtle effects compared to high‐severity fire and drought‐related tree mortality (McGinn, Jones, et al., 2025; Ng et al., 2025). Balancing the trade‐offs between ecological disturbance‐related habitat loss and the effects of management actions necessary to mitigate such losses is crucial for effective adaptive management and species conservation. In conclusion, large‐scale passive acoustic surveys analyzed within a dynamic occupancy framework can be a robust and cost‐effective approach for biodiversity monitoring in an era of rapid ecological change.

## AUTHOR CONTRIBUTIONS

Jason M. Winiarski, Connor M. Wood, Holger Klinck, and M. Zachariah Peery conceived the study. Jason M. Winiarski, Sheila A. Whitmore, Jonathan P. Eiseman, Erin C. Netoskie, Matthias E. Bieber, H. Anu Kramer, and Kevin G. Kelly collected and managed the data. Stefan Kahl developed the BirdNET algorithm and processed the raw audio recordings. Jason M. Winiarski analyzed the data with input from M. Zachariah Peery. Jason M. Winiarski and M. Zachariah Peery wrote the manuscript, and all authors contributed critically to the drafts and gave final approval for publication.

## CONFLICT OF INTEREST STATEMENT

The authors declare no conflicts of interest.

## Supporting information


Appendix S1:

## Data Availability

Complete locational information of survey grid cells and associated spotted owl detections are sensitive; these data are owned by the Department of Forest and Wildlife Ecology at the University of Wisconsin‐Madison and are available to qualified researchers by contacting the principal investigator of the Sierra Nevada Acoustic Monitoring Program (M. Zachariah Peery; email: mpeery@wisc.edu) and requesting access. Raw audio data can be downloaded upon request from the Sierra Nevada Bioacoustic Monitoring Data Hub (https://acousticdownload.russell.wisc.edu). Anonymized encounter histories (Winiarski et al., 2025) that were used in the occupancy analysis are available from Zenodo at https://doi.org/10.5281/zenodo.17591080.
