# Supplementary material for: Passive acoustic monitoring can provide insights into occupancy dynamics and impacts of disturbance for at‐risk species
Source: Ecol Appl. 2026 Jan 15;36(1):e70177. doi: 10.1002/eap.70177 (PMC12808558; doi:10.1002/eap.70177)
Supplement: Supplementary file 1 — Appendix S1: [file EAP-36-e70177-s001.pdf]

Appendix S1

Passive acoustic monitoring can provide insights into occupancy dynamics and impacts of disturbance for at-risk species

Jason M. Winiarski, Sheila A. Whitmore, Connor M. Wood, Jonathan P. Eiseman, Erin C. Netoskie, Matthias E. Bieber, H. Anu Kramer, Kevin G. Kelly, Kate McGinn, Craig Thompson, Sarah C. Sawyer, Stefan Kahl, Holger Klinck, and M. Zachariah Peery

Ecological Applications

TABLES

TABLE S1 Justification for environmental variables used in the California spotted owl dynamic occupancy model fit to ARU data from 2021–2024.

Predictor	Submodel(s)	Hypothesis	Rationale
Latitude	Initial occupancy	California spotted owl occupancy (which is highly correlated with abundance) is highest in the central Sierra Nevada (non-linear relationship with elevation).	Species tend to have higher abundance or occupancy near the center of their geographic range.[1]
Elevation	Initial occupancy	California spotted owl occupancy is highest at mid-elevations (non-linear relationship with elevation).	Most California spotted owl habitat in the Sierra Nevada is concentrated in mid-elevation forests.[2]
Canopy height	Initial occupancy	California spotted owl occupancy has a positive linear relationship with canopy height.	California spotted owls are more likely to occupy sites with greater canopy height, and owl nest sites occur in areas with patches of tall trees.[3,4]
Proportion of a cell burned by high-severity wildfire	Initial occupancy, colonization, and extinction	California spotted owl occupancy and colonization has a negative linear relationship with the proportion of a cell burned at high-severity; extinction has a positive linear relationship with high-severity fire.	Large, severe fires have detrimental effects on occupancy dynamics and threaten the persistence of spotted owl populations.[5,6]

[1]Brown, J. H. 1984. "On the relationship between abundance and distribution of species." *The American Naturalist* 124:255–279.

[2]Gutiérrez, R. J., P. N. Manley, P. A. Stine. 2017. "*The California spotted owl: current state of knowledge*." Gen. Tech. Rep. PSW-GTR-254. U.S. Department of Agriculture, Forest Service, Pacific Southwest Research Station, Albany, CA.

[3]Kramer, H. A., K. G. Kelly, S. A. Whitmore, W. J. Berigan, D. S. Reid, C. M. Wood, H. Klinck, S. Kahl, P. N. Manley, S. C. Sawyer, and M. Z. Peery. 2024. "Using bioacoustics to enhance the efficiency of spotted owl surveys and facilitate forest restoration." *Journal of Wildlife Management* 88:p.e22533.

[4]North, M. P., J. T. Kane, V.R. Kane, G. P. Asner, W. J. Berigan, D. J. Churchill, S. Conway, R. J. Gutiérrez, S. Jeronimo, J. J. Keane, and A. Koltunov. 2017. "Cover of tall trees best predicts California spotted owl habitat." *Forest Ecology and Management* 405:166–178.

[5]Jones, G. M., R. J. Gutiérrez, D. J. Tempel, S. A. Whitmore, W. J. Berigan, and M. Z. Peery. 2016. "Megafires: an emerging threat to old-forest species." *Frontiers in Ecology and the Environment* 14:300–306.

[6]Jones, G. M., H. A. Kramer, W. J. Berigan, S. A. Whitmore, R. J. Gutiérrez, and M. Z. Peery. 2021. "Megafire causes persistent loss of an old-forest species." *Animal Conservation* 24:925–936.

TABLE S2 Mean, standard deviation (SD), 95% credible intervals, and convergence diagnostics ($\hat{R}$ and effective sample size; ESS) of parameter estimates from California spotted owl dynamic occupancy model fit to ARU data from 2021–2024.

Submodel	Parameter	Mean	SD	2.5%	97.5%	Rhat	ESS
Initial occupancy	Intercept	-0.497	0.391	-1.262	0.305	1.00	1031
	Elevation	-0.239	0.117	-0.487	-0.019	1.00	3496
	Elevation^2	-0.429	0.096	-0.619	-0.244	1.00	3054
	Latitude	-0.893	0.301	-1.495	-0.298	1.00	1397
	Latitude^2	-0.209	0.183	-0.576	0.138	1.00	2262
	Canopy height	1.137	0.126	0.895	1.387	1.00	3470
	High severity burn	-0.193	0.112	-0.42	0.018	1.00	3456
	σ_{Forest}	0.807	0.322	0.383	1.631	1.00	1566
Colonization	Intercept	-2.043	0.228	-2.469	-1.567	1.00	1244
	Year (2022)	0.28	0.187	-0.08	0.649	1.00	3126
	Year (2023)	-0.362	0.227	-0.805	0.077	1.00	3258
	High severity burn	-0.77	0.113	-1.003	-0.555	1.00	3339
	σ_{Forest}	0.417	0.203	0.123	0.911	1.00	1264
Extinction	Intercept	-0.362	0.258	-0.868	0.14	1.00	883
	Year (2022)	-0.126	0.227	-0.548	0.322	1.00	2939
	Year (2023)	-0.153	0.239	-0.631	0.301	1.00	2692
	High severity burn	0.73	0.14	0.466	1.015	1.00	4728
	σ_{Forest}	0.553	0.246	0.213	1.172	1.00	1351
Detection	Intercept	0.507	0.151	0.198	0.799	1.01	616
	Year (2022)	0.006	0.091	-0.175	0.184	1.00	2428
	Year (2023)	0.047	0.092	-0.127	0.237	1.00	2101
	Year (2024)	-0.168	0.098	-0.36	0.02	1.00	2566
	Date	0.013	0.034	-0.052	0.076	1.00	3789
	Date^2	-0.04	0.026	-0.091	0.012	1.00	3117
	log(ARU hours)	0.582	0.033	0.518	0.648	1.00	3550
	σ_{Forest}	0.325	0.131	0.156	0.667	1.00	1232

TABLE S3 California spotted owl occupancy (ψ) estimates for the Sierra Nevada region, by forest, and year. Mean, standard deviation, and 95% credible intervals of ψ are shown.

**National Forest**	**Year**	**Mean**	**SD**	**2.5%**	**97.5%**
Lassen	2021	0.196	0.033	0.136	0.267
	2022	0.157	0.024	0.114	0.206
	2023	0.175	0.028	0.124	0.234
	2024	0.146	0.029	0.093	0.208
Plumas	2021	0.182	0.029	0.129	0.242
	2022	0.211	0.023	0.168	0.255
	2023	0.261	0.028	0.208	0.317
	2024	0.239	0.029	0.187	0.298
Tahoe	2021	0.362	0.036	0.292	0.433
	2022	0.349	0.032	0.289	0.414
	2023	0.370	0.038	0.298	0.446
	2024	0.339	0.040	0.261	0.417
Eldorado	2021	0.414	0.039	0.339	0.491
	2022	0.320	0.031	0.262	0.384
	2023	0.315	0.033	0.251	0.385
	2024	0.271	0.035	0.205	0.343
Stanislaus	2021	0.443	0.040	0.366	0.523
	2022	0.447	0.036	0.377	0.519
	2023	0.481	0.042	0.402	0.564
	2024	0.459	0.048	0.371	0.555
Sierra	2021	0.178	0.030	0.120	0.242
	2022	0.215	0.025	0.167	0.265
	2023	0.265	0.031	0.206	0.327
	2024	0.244	0.033	0.183	0.312
Sequoia	2021	0.419	0.046	0.328	0.505
	2022	0.311	0.037	0.242	0.386
	2023	0.295	0.037	0.224	0.370
	2024	0.258	0.039	0.187	0.341
All forests	2021	0.299	0.014	0.272	0.326
	2022	0.279	0.015	0.249	0.309
	2023	0.303	0.016	0.273	0.333
	2024	0.275	0.016	0.243	0.307

TABLE S4 Estimates of net change in occupancy ($\bar{\lambda}$) for the Sierra Nevada region and by national forest. Mean, standard deviation, and 95% credible intervals of $\bar{\lambda}$ are shown, along with probability of a declining or stable/increasing trend.

National Forest	Mean	SD	2.5%	97.5%	Probability of trend
Lassen	0.92	0.08	0.77	1.07	0.85
Plumas	1.11	0.08	0.98	1.28	0.94
Tahoe	0.98	0.05	0.89	1.08	0.65
Eldorado	0.87	0.04	0.79	0.96	1.00
Stanislaus	1.01	0.04	0.93	1.10	0.62
Sierra	1.13	0.08	0.98	1.32	0.95
Sequoia	0.86	0.05	0.77	0.95	1.00
All forests	0.98	0.02	0.93	1.02	0.85

TABLE S5 Summary of annual personnel, data storage, and analysis needs for the Sierra Nevada Passive Acoustic Monitoring Program (2021–2024).

**Resource**	**Purpose**	**Quantity**	**Notes**
Seasonal staff	Deploy and retrieve autonomous recording units	~10 field technicians per season	Technicians work ~40 hours per week from early April–August
Research staff	Hire field technicians, coordinate fieldwork, manually vet putative spotted owl detections, and manage/process acoustic data	4 researchers (3 field crew leaders and 1 data scientist)	Full-time, year-round positions
Data storage	Archive deployment metadata, raw audio data, and associated BirdNET outputs	~100 terabytes of raw audio (FLAC files) and ~5 terabytes of BirdNET predictions (JSON files) per year[1,2]	UW-Madison ResearchDrive (short-term; <8 months) and UW-Madison S3 Drive (long-term)[3,4]
Computing	Generate BirdNET predictions per 3-second audio segments in each FLAC file	~3 weeks of processing time per year	Amazon Web Services used for cloud computing

[1]Audio recorded by Swift autonomous recording units are stored onboard the unit on an SD card in WAV file format. To compress audio (without changing sound quality or data), WAV files are converted and stored as FLAC files (resulting in a ~50% reduction in file size).
[2]Our customized version of BirdNET generates a compressed JSON file (json.gz) containing predictions of 241 species for each 3-second segment of audio in a corresponding FLAC file. From 2023–onward, we included embeddings in each JSON, increasing file size by ~20%.
[3]It is half the cost to store data on S3, but it must be downloaded from S3 in order to be analyzed.
[4]Data migration between storage locations and Amazon Web Services performed with Globus file transfer system

FIGURES

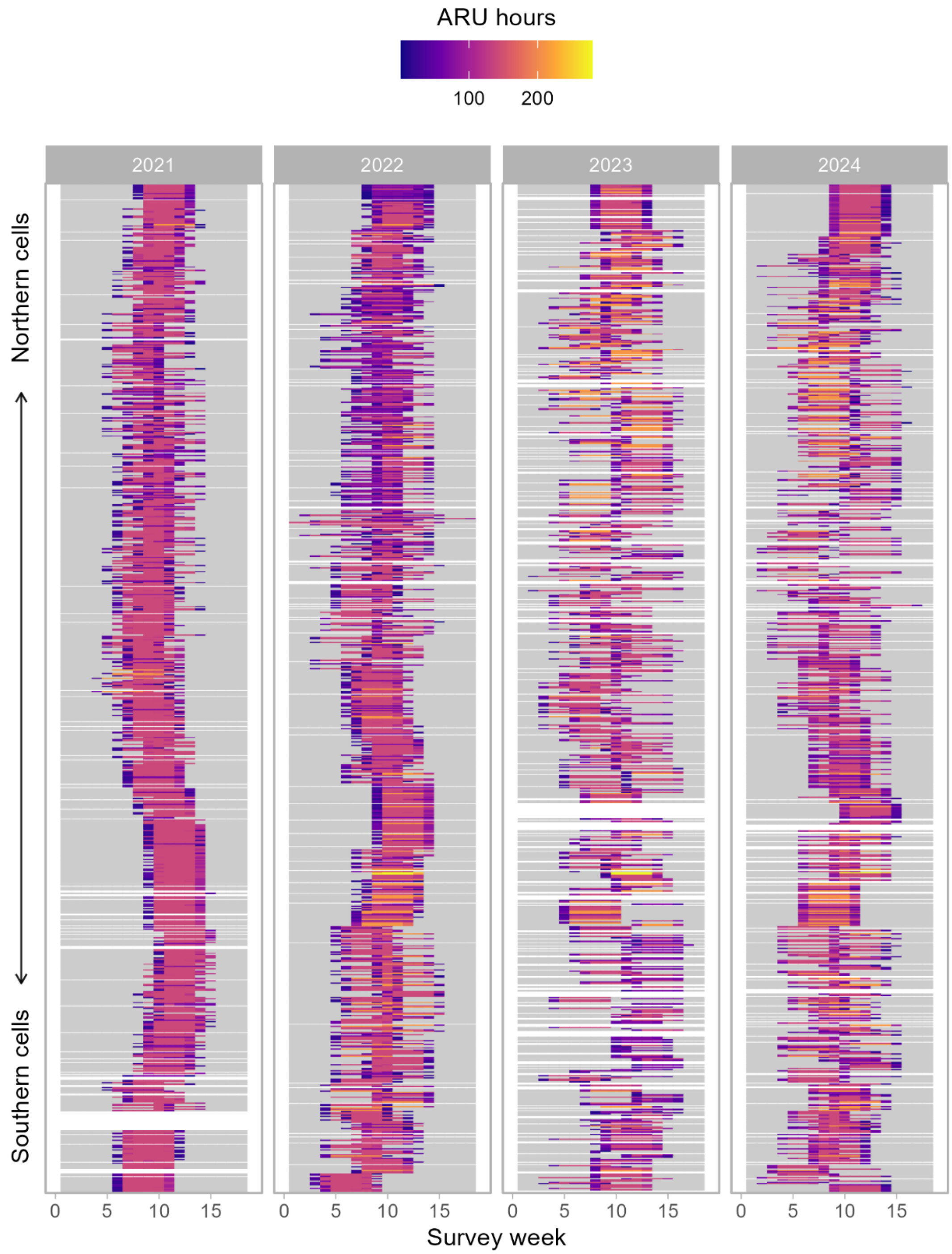

FIGURE S1 Passive acoustic survey effort and timing by hexagonal grid cell in the Sierra Nevada region, 2021–2024. Cells are ordered by latitudinal position, with warmer colors indicating greater survey effort, and gray shading indicating weeks without survey effort. Missing rows show cells that were not surveyed in one or more years.

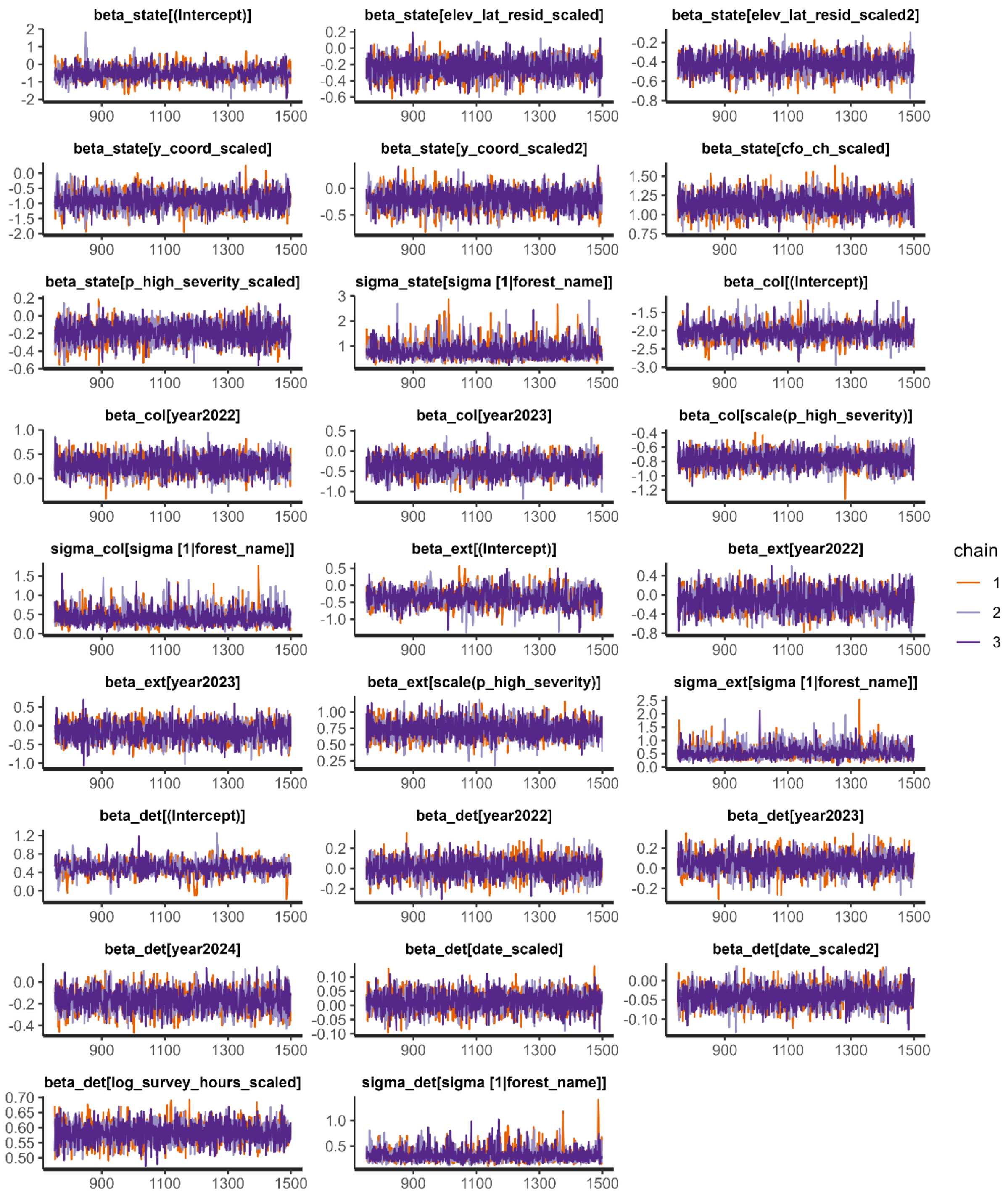

FIGURE S2 Traceplots for the California spotted owl dynamic occupancy model.

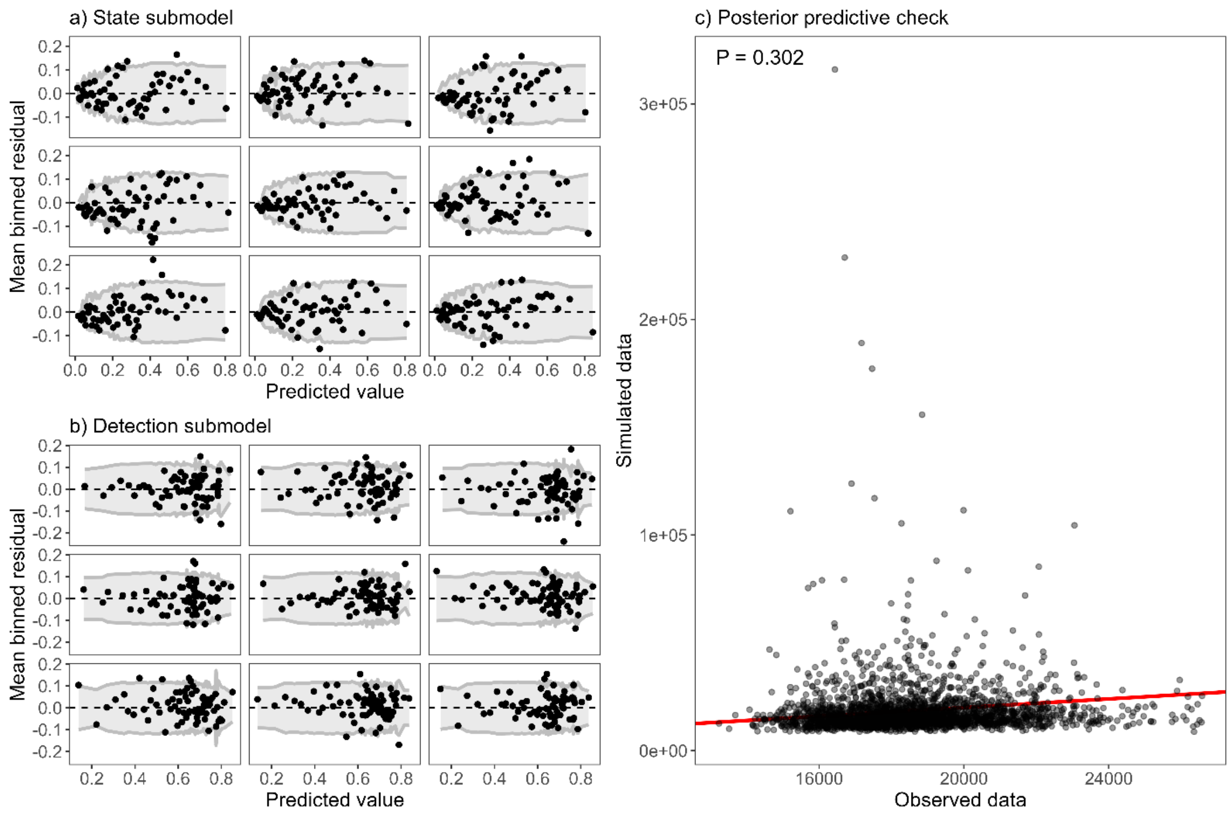

FIGURE S3 Residual plots for state (a) and detection (b) submodels and posterior predictive check plot (c) for California spotted owl dynamic occupancy model. Goodness-of-fit test (Bayesian *p*-value = 0.302) and posterior predictive check plot indicated adequate model fit.

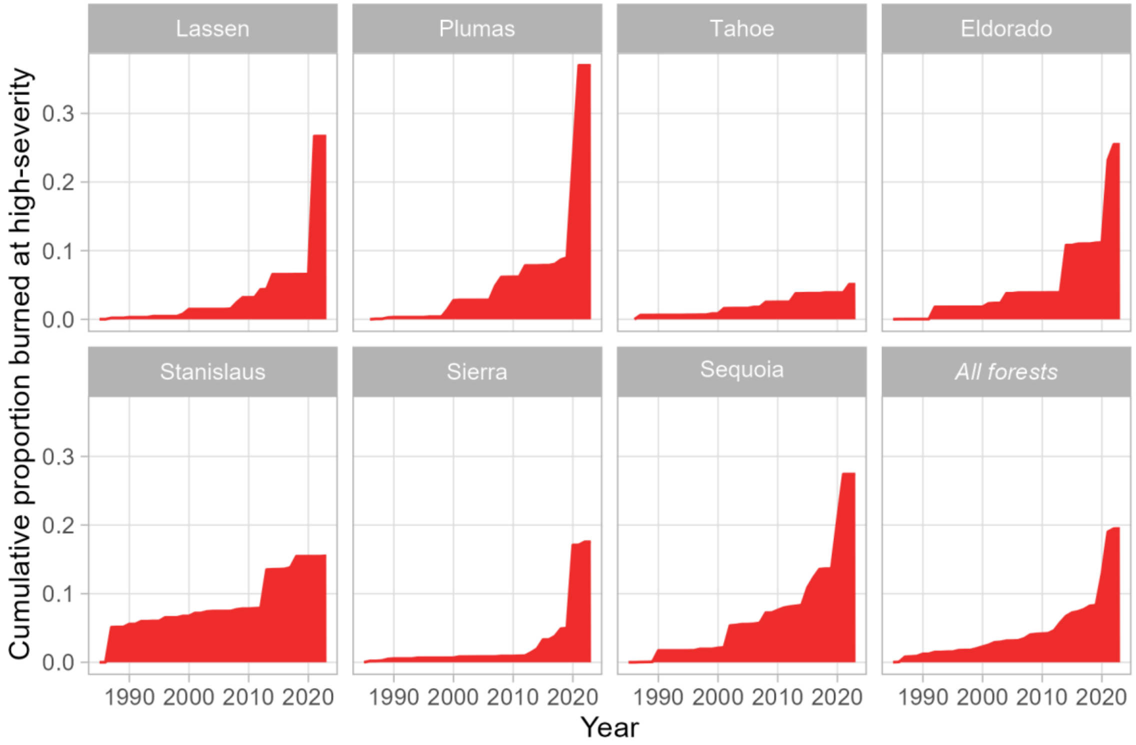

FIGURE S4 Cumulative proportion burned at high-severity (red polygon) of each national forest within the boundary of the Sierra Nevada passive acoustic monitoring program, 1985–2023.

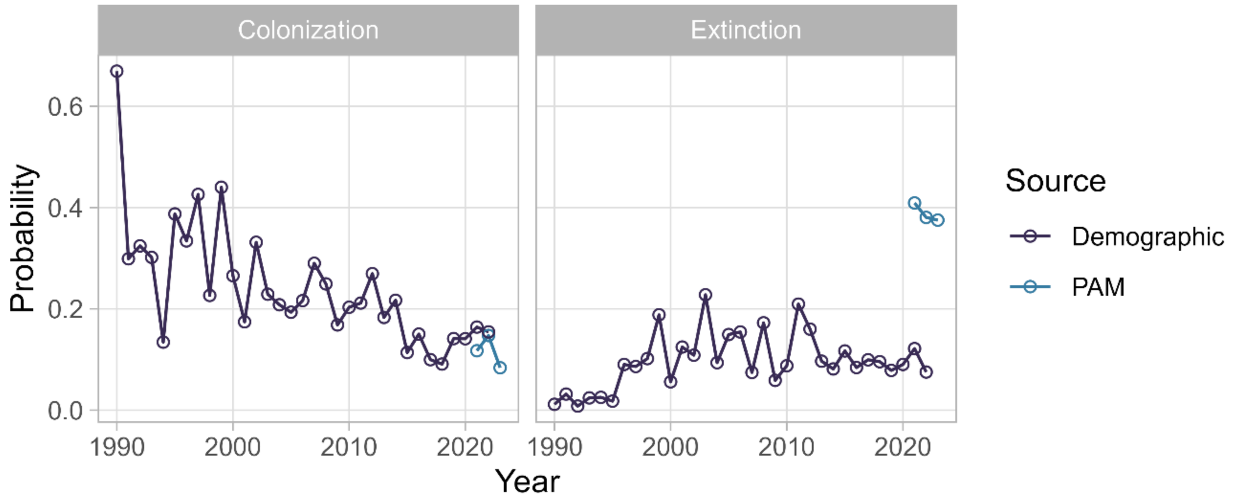

FIGURE S5 California spotted owl colonization (left panel) and extinction (right panel) probabilities by year derived from our passive acoustic monitoring program (blue) and a recent meta-analysis from demographic study areas in the Sierra Nevada, California, USA (purple).
